# Meat and meat products as potential sources of emerging MDR *Bacillus cereus*: *gro*EL gene sequencing, toxigenic and antimicrobial resistance

**DOI:** 10.1186/s12866-024-03204-9

**Published:** 2024-02-07

**Authors:** Abdelazeem M. Algammal, Hamza M. Eid, Saad Alghamdi, Hanaa Ghabban, Roba Alatawy, Enas A. Almanzalawi, Tahani M. Alqahtani, Sabreen G. Elfouly, Gihan M. Mohammed, Helal F. Hetta, Reham M. El-Tarabili

**Affiliations:** 1 Department of Bacteriology, Immunology and Mycology, Faculty of Veterinary Medicine, Suez Canal University, Ismailia, 41522 Egypt; 2 Department of Microbiology, Faculty of Veterinary Medicine, Ain Shams University, Cairo, 11566 Egypt; 3 Department of Clinical Laboratory Sciences, Faculty of Applied Medical Sciences, Umm Al-Qura University, Makkah, 24381 Saudi Arabia; 4 Department of Biology, Faculty of Science, University of Tabuk, Tabuk, 71491 Saudi Arabia; 5 Medical Microbiology Department, School of Medicine, University of Tabuk, Tabuk, 71491 Saudi Arabia; 6 Department of Biological Sciences, Faculty of Sciences, King Abdulaziz University, Jeddah, 21589 Saudi Arabia; 7 Department of Bacteriology, Animal Health Research Institute, Port-Said branch, Port Said, 42511 Egypt; 8 Department of Medical Microbiology and Immunology, Faculty of Medicine, Assiut University, Assiut, 71515 Egypt

**Keywords:** MDR *B. cereus*, Meat and meat products, Resistance patterns, Toxigenic genes, Resistance genes

## Abstract

**Background:**

*Bacillus cereus* is implicated in severe foodborne infection in humans. This study intended to assess the occurrence, *gro*EL gene sequencing, biofilm production, and resistance profiles of emerged multidrug resistant (MDR) *B. cereus* in meat and meat product samples. Moreover, this work highlights the virulence and toxigenic genes (*hbl*ABCD complex, *nhe*ABC complex, *cyt*K, *ces*, and *pc-plc*) and antimicrobial resistance genes (*bla*1, *tet*A, *bla*2, *tet*B, and *erm*A).

**Methods:**

Consequently, 200 samples (sausage, minced meat, luncheon, beef meat, and liver; *n* = 40 for each) were indiscriminately collected from commercial supermarkets in Port Said Province, Egypt, from March to May 2021. Subsequently, food samples were bacteriologically examined. The obtained isolates were tested for *gro*EL gene sequence analysis, antibiotic susceptibility, biofilm production, and PCR screening of toxigenic and resistance genes.

**Results:**

The overall prevalence of *B. cereus* among the inspected food samples was 21%, where the highest predominance was detected in minced meat (42.5%), followed by beef meat (30%). The phylogenetic analysis of the *gro*EL gene exposed that the examined *B. cereus* strain disclosed a notable genetic identity with other strains from the USA and China. Moreover, the obtained *B. cereus* strains revealed β-hemolytic activity, and 88.1% of the recovered strains tested positive for biofilm production. PCR evidenced that the obtained *B. cereus* strains usually inherited the *nhe* complex genes (*nhe*A and *nhe*C: 100%, and *nhe*B: 83.3%), followed by *cyt*K (76.2%), *hbl* complex (*hbl*C and *hbl*D: 59.5%, *hbl*B: 16.6%, and *hbl*A: 11.9%), *ces* (54.7%), and *pc-plc* (30.9%) virulence genes. Likewise, 42.9% of the examined *B. cereus* strains were MDR to six antimicrobial classes and encoded *bla*1, *bla*2, *erm*A, and *tet*A genes.

**Conclusion:**

In summary, this study highlights the presence of MDR *B. cereus* in meat and meat products, posing a significant public health risk. The contamination by *B. cereus* is common in minced meat and beef meat. The molecular assay is a reliable fundamental tool for screening emerging MDR *B. cereus* strains in meat and meat products.

## Background

Communicable diarrheal diseases are incriminated in significant morbidity and mortalities all over the world [1]. *Bacillus cereus* (*B. cereus*) is one the utmost predominant food-borne pathogens, resulting in severe food poisoning. The disease is mainly associated with severe diarrhea, vomiting, liver failure, abdominal pain, and necrotic enteritis [2]. Moreover, *B. cereus* is reported frequently as the most prevailing pathogen found in several food products, including meat and meat products, milk products, and food of plant origin, which is considered a public health threat [3].

*B. cereus* is a Gram-positive, motile, and spore-forming rod. *B. cereus*, a ubiquitous pathogen, is widely distributed in food, soil, water, and plants [4]. The pathogenicity of *B. cereus* is endorsed mainly by various virulence factors and toxins encoded by the corresponding genes. The infection takes place as a result of the ingestion of contaminated food with *B. cereus.* The cells of *B. cereus* attached to the host intestinal mucosa then undergo colonization, followed by enterotoxin production [5, 6]. The most common virulence factors associated with *B. cereus* infection include the heat-labile enterotoxins group: 1-cytotoxin K (regulated by the *cyt*K gene), 2-non-hemolytic enterotoxin (encoded by the *nhe*ABC gene complex), and 3-hemolysin BL (encoded by the *hbl*ABCD gene complex), the cereulide toxin (regulated by the *ces* gene), and phospholipase C (regulated by the *pc*-*plc* gene) [7–9]. Several food poisoning outbreaks triggered by *B. cereus* were reported globally [10]. The disease is associated with two clinical forms: diarrheal and emetic forms. Cytotoxin K, a potent enterotoxin, is considered the main virulence determinant incriminated in severe diarrhea. Besides, the heat-labile enterotoxins are responsible for the diarrheal syndrome. Moreover, the emetic syndrome is ascribed mainly to the cereulide toxin [6, 11, 12]. Furthermore, *B. cereus* is incriminated in severe infections in humans, including pneumonia [13], bacteremia in neonates [5], gas gangrene, bacterial meningitis, and eye infections [14].

The biofilm production is commonly associated with *B. cereus* to enhance its survival in adverse environmental circumstances. Biofilms produced by *B. cereus* are considered the principal source of device contamination during food processing [15]. Spore production by *B. cereus* makes biofilm difficult to eliminate due to the resistance of spores to radiation and heat [16].

Antimicrobial resistance is markedly raised all over the world, suggesting a public health hazard [17]. *B. cereus* inherited several antibiotic-resistance genes that enable the bacteria to resist several antimicrobial classes [6]. *B. cereus* is frequently resistant to antibiotics belonging to the β-lactam class [18, 19]. Moreover, *B. cereus* could display acquired resistance to some antibiotics such as streptomycin, tetracycline, and erythromycin [20]. A previous molecular investigation emphasized that *B. cereus* harbored resistance genes against macrolides, tetracycline, and β-lactam antibiotics [6]. The indiscriminate use of antimicrobial agents consequences the development of multidrug-resistant pathogens [21, 22].

Herein, we intended to determine the prevalence, *gro*EL gene sequencing, biofilm production, and resistance profiles of emerged MDR *B. cereus* in meat and its products. Moreover, this work highlights the virulence and toxigenic genes (*hbl*ABCD complex, *nhe*ABC complex, *cyt*K, *ces*, and *pc-plc*) and antimicrobial resistance genes (*bla*1, *tet*A, *bla*2, *tet*B, and *erm*A) inherited and acquired by the obtained isolates.

## Materials and methods

### Sampling

Two hundred samples (sausage, minced meat, luncheon, beef meat, and liver; *n* = 40 for each) were indiscriminately collected from commercial supermarkets in Port-Said Province, Egypt, from March to May 2021. Consequently, samples were transported as soon as possible to the laboratory.

### Isolation, identification, and enumeration of *B. cereus*

About 25 g of each collected sample was homogenized in 225 mL of tryptic soy broth (BD Difco, USA) using a bag-mixer stomacher followed by serial dilution. Afterward, 0.1 ml of each diluted specimen was spread evenly with a spreader onto Mannitol Egg-Yolk Polymyxin (MYP) (BD Difco, USA) agar plates and left incubated at 37 °C for 24 h [23]. Typical *B. cereus* colonies were pink and bounded by a zone of precipitation, specifying the lecithinase activity. Then, colonies were enumerated with a colony counter. *B. cereus* was identified as consistent with culture and morphological characteristics, endospores, and biochemical tests (catalase, indole, H_2_S production, oxidase, methyl red, sugar fermentation, and Voges-Proskauer tests), according to Maturin and Peeler [24]. The identity of isolates was determined genetically using specific primers of *gro*EL gene, as previously described [25].

### *gro*EL gene sequencing

Herein, the retrieved *B. cereus* isolates revealed similar phenotypic characteristics; therefore, a PCR product of one indiscriminately chosen isolate was used for *gro*EL sequencing in both directions (QIAGEN Sciences Inc., Germantown, MD, USA). The Bigdye Terminator V3.1 cycle sequencing kit was used in gene sequencing (Thermo Fisher Scientific, Waltham, MA, USA). Besides, the phylogenetic tree was assembled in accordance with the neighbor-joining approach using the MEGA 11 software [26].

### Hemolytic activity and biofilm production

The retrieved strains were streaked on 5% sheep blood agar (BD Difco, USA) and incubated at 24 °C for 48 h. The occurrence of β-hemolysis specifies the positive result, according to Wiwat and Thiramanas [27].

Using the microtitre plate method, a 200 µl suspension of *B. cereus* (10^8^ CFU/ mL) in tryptone soy broth (Difco, USA) was added to a 96-well polystyrene microtiter plate. The bacterial cell suspension was incubated at 30 °C for 24 h. Subsequently, plates were decanted, washed, dried for 30 min, and stained with crystal violet for 15 min. After staining, the plates were decanted, washed, and dried for 15 min. For each well, 150 µL of ethanol (95%) was added, and plates were left for 30 min. The absorbance was estimated at 595 nm using a microplate reader (un-inoculated wells included as negative controls). Besides, *B. cereus* ATCC 10876 was involved as a positive control. The tested isolates were classified in consistent their OD value into strong (4 x ODc < ODs), moderate (2 x ODc < ODs ≤ 4 x ODc), weak (ODc < ODs ≤ 2 x ODc), and non (ODs ≤ ODc) biofilm producers [28].

### Antimicrobial susceptibility testing

The antibiotic susceptibility of the obtained *B. cereus* strains was assessed on Mueller-Hinton agar (BD Dico, USA) using the disc diffusion test consistent with Park et al. [29]. Eleven antimicrobial discs were involved including erythromycin (E, 15 µg), ampicillin (AMP/ 10 µg), streptomycin (S/ 10 µg), amoxicillin (AMX/ 30 µg), sulfamethoxazole/trimethoprim (SXT/ 25 µg), cefepime (FEP/ 30 µg), ceftriaxone (CRO/ 30 µg), amoxicillin-clavulanic acid (AMC/ 30 µg), tetracycline (TE/ 30 µg), vancomycin (VA/ 30 µg), and levofloxacin (LEV/ 5 µg) (Oxoid, UK). The tested antimicrobial agents are commonly used in the veterinary and health sectors in Egypt. The interpretation of results was carried out as consistent with CLSI, 2017 [30]. *S. aureus* ATCC 29213 was involved as a control strain. Also, the obtained *B. cereus* strains were classified into MDR according to Magiorakos et al. [31]. Besides, the MAR (multiple antibiotic resistance) index was assessed consistent with Krumperman [32].

### Detection of toxigenic and antibiotic resistance genes

Detection of virulence-related (*hbl*ABCD, *nhe*ABC, *cyt*K, *ces*, and *pc-plc*) and antibiotic resistance genes (*tet*A, *bla*1, *erm*A, *tet*B, and *bla*2) was performed using PCR. The extraction of genomic DNA was performed by the QIAamp DNA Mini Kit (QIAGEN Sciences Inc., Germantown, USA). The bacterial pellet was mixed with 180 µL Buffer AL in a 1.5 ml microcentrifuge tube and incubated at 70 °C for 10 min. Subsequently, 200 µL of was added, mixed by pulse-vortex for 15 s, and centrifuged to remove drops from inside the lid of the collecting tube. The mixture was transferred to the spin column and centrifuged at 6500 x g for 1 min. The spin column was then moved to a 2 mL Eppendorf tube and washed multiple times with 500 µL of Buffer AW1 and AW2 at 6,000–20,000 x g for 1–3 min. The extracted DNA was collected in a new collecting tube by adding 200 µL of elution Buffer AE. Moreover, DNA-free reactions were used as negative controls, while reference *B. cereus* strains (The AHRI, Dokki, Egypt) were used as positive controls. Table 1 illustrates the used primer sequences.

**Table 1:** Illustrates the oligonucleotide sequences used in PCR assay and their specific amplicons

	Primers	Sequences	Amplicons (bp)	References
Confirmatory gene Chaperonin protein	*groEL*	F:TGCAACTGTATTAGCACAAGC T R:TACCACGAAGTTTGTTCACTACT	533	[25]
*hbl complex*	*hbl*A	F: AAGCAATGGAATACAATGGG R: AGAATCTAAATCATGCCACTGC	1154	[33]
	*hbl*B	F: AAGCAATGGAATACAATGGG R: AATATGTCCCAGTACACCCG	2684	
	*hbl*C	F:GATACTCAATGTGGCAACTGC R:TTGAGACTGCTCGTCTAGTTG	740	
	*hbl*D	F:ACCGGTAACACTATTCATGC R:GAGTCCATATGCTTAGATGC	829	
*nhe complex*	*nhe*A	F:GTTAGGATCACAATCACCGC R:ACGAATGTAATTTGAGTCGC	755	
	*nhe*B	F:TTTAGTAGTGGATCTGTACGC R:TTAATGTTCGTTAATCCTGC	743	
	*nhe*C	F: TGGATTCCAAGATGTAACG R: ATTACGACTTCTGCTTGTGC	683	
Enterotoxin	*cyt*K	F: ACA GAT ATC GGI CAA AAT GC R: CAA GTI ACT TGA CCI GTT GC	421	[34]
Emetic toxin	*ces*	F:GGTGACACATTATCATATAAGGTG R:GTAAGCGAACCTGTCTGTAACAACA	1271	
Phospholipase C	*pc-plc*	F:GAGTTAGAGAACGGTATTTATGCTGC R: CTACTGCCGCTCCATGAATCC	411	[35]
Resistance genes	*bla*1	F: CATTGCAAGTTGAAGCGAAA R:TGTCCCGTAACTTCCAGCTC	680	[36]
	*bla*2	F: TTGTCGATTCTTCTTGGGATG R: CCCCTACTTCTCCATGACCA	483	
	*tet*A	F: GGCGGTCTTCTTCATCATGC R: CGGCAGGCAGAGCAAGTAGA	502	[37]
	*tet*B	F:CATTAATAGGCGCATCGCTG R:TGAAGGTCATCGATAGCAGG	930	
	*erm*A	F: TCTAAAAAGCATGTAAAAGAA R:CTTCGATAGTTTATTAATATTAGT	645	[38]

### Statistical analyses

G*Power (3.1.9.7) was used to evaluate sample size using Effect size f = 0.25 and power (1-β err prob = 0.80). Categorical variables were presented as frequencies and percentages. Associations between categorical variables were determined using the chi-square test (SAS software, version 9.4, SAS Institute, Cary, NC, USA) (*p*-value < 0.05 points to a significant variance). The “cor” function in the R-software (version 4.0.2; https://www.r-project.org/) and the “corrplot” package were used to create a correlation matrix for resistance profiles and resistance genes, as well as biofilm production and antimicrobial resistance. The heatmap of antimicrobial resistance patterns was performed using the GraphPad Software (version 8.0.1).

## Results

### Phenotypic features and the frequency of *B. cereus* isolates

Herein, the prevalence of *B. cereus* in the inspected food samples was 21% (42/200). The highest prevalence was noticed in minced meat (42.5%; 17/40), followed by beef meat (30%; 12/40), sausage (12.5%; 5/40), luncheon (10%; 4/40), and liver (10%; 4/40), as demonstrated in Table 2. Statistically, there is a significant variance in the frequency of *B. cereus* in various inspected food samples (*p* < 0.05).

**Table 2:** The prevalence and total count of *B. cereus* in the inspected food products

Types of samples	Isolates		Mean + SE CFU/g	MIN	MAX
	n	%			
Minced meat	17	42.5	11.75 × 10^3^±9.42 × 10^3^	1.5 × 10^3^	4 × 10^4^
Beef meat	12	30	3 × 10^3^±3.43 × 10^2^	1 × 10^3^	5 × 10^3^
Sausage	5	12.5	2.87 × 10^3^±4.26 × 10^2^	2 × 10^3^	4 × 10^3^
Luncheon	4	10	2.56 × 10^3^±4.57 × 10^2^	1 × 10^3^	4.5 × 10^3^
Liver	4	10	2.1 × 10^3^±5.56 × 10^2^	1 × 10^3^	4 × 10^3^
Total	42	21			
*Chi square* *P* value	16.333 0.002603				

The obtained isolates exhibited the distinctive morphological features of *B. cereus*, where all the isolates were Gram-positive, motile, short bacilli with non-bulging endospores. On MYP media, colonies were pink with a characteristic precipitation zone (lecithinase positive). Moreover, the obtained isolates tested positive for Voges-Proskauer, catalase, citrate utilization, glucose fermentation, and nitrate reduction. Likewise, the isolates tested negative for oxidase, H_2_S production, methyl red, indole, and mannitol fermentation. Besides, all the obtained isolates inherited the *gro*EL gene.

In this study, the mean of *B. cereus* count was 11.75 × 10^3^, 3 × 10^3^, 2.87 × 10^3^, 2.56 × 10^3^, and 2.1 × 10^3^ CFU/g in the examined minced meat, beef meat, sausage, luncheon, and liver samples, respectively (Table 2).

### Phylogenetic analysis of the *gro*EL gene

**Fig. 1:**
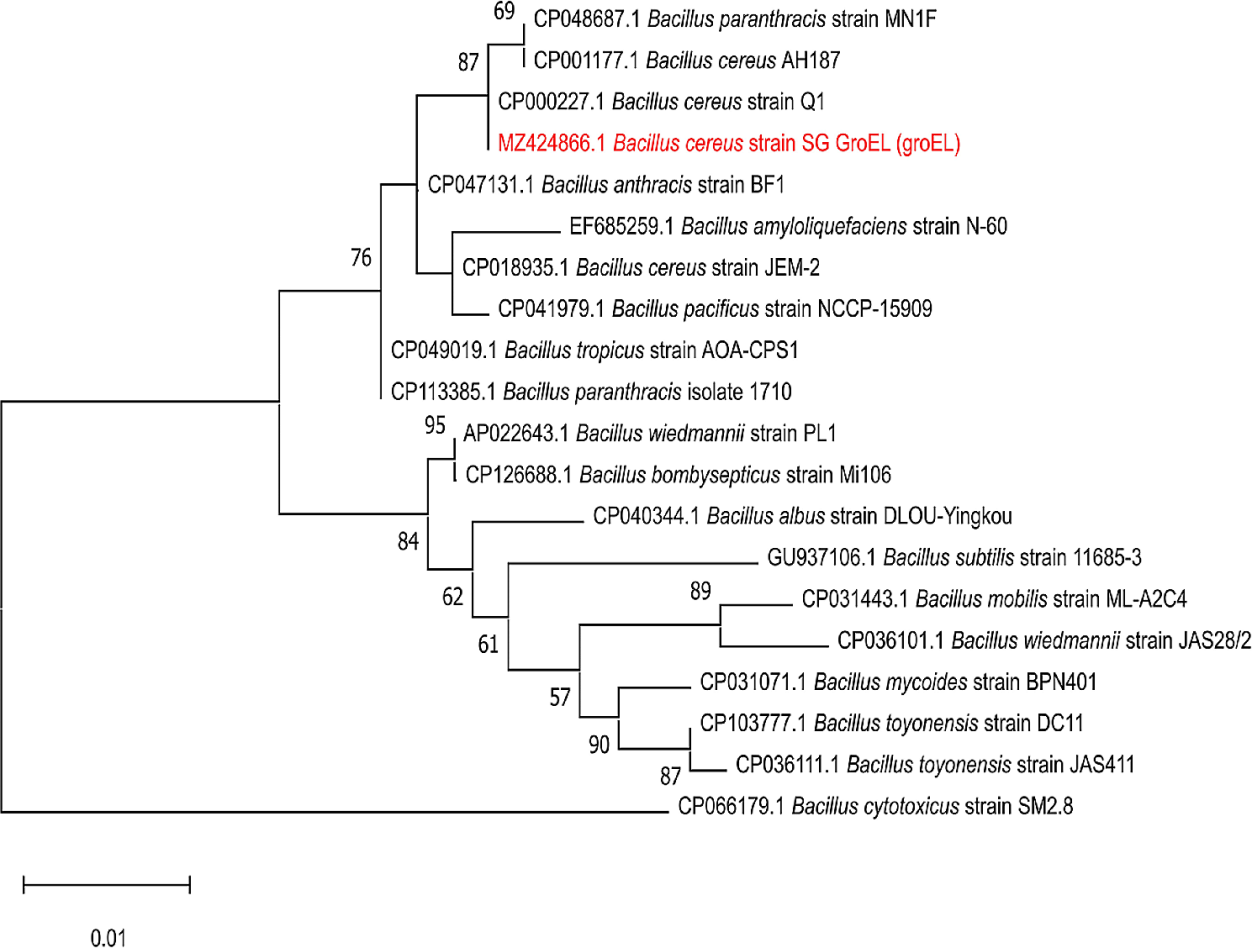
This figure clarifies the phylogenetic analyses of *gro*EL sequencing. The tree illuminates the genetic similarity among the selected *B. cereus* strain in this study and other strains acquiesced in the GenBank database. The tree was generated through bootstrap analysis with 1000 replicates, and the results are depicted above the branches

The phylogenetic analysis confirmed that the examined *B. cereus* strain (Accession number: MZ424866) revealed a notable genetic similarity with other isolates from various geographical areas. For instance, *B. cereus* strain Q1 (100%) of China (Accession number: CP000227), strain AH187 (99.79%) of the USA (Accession number: CP001177), and *B. cereus* strain JEM-2 (99.36%) isolated from the USA (Accession number: CP018935), as illustrated in Fig. 1.

### Hemolysis and biofilm production ability

**Fig. 2:**
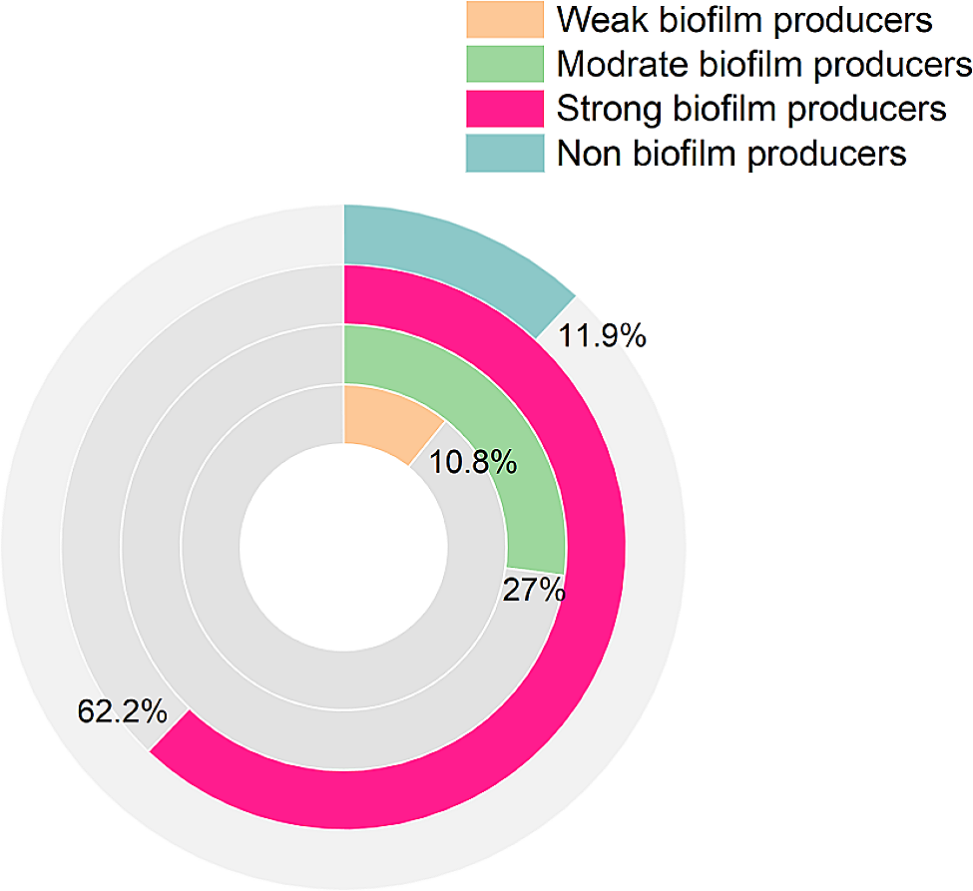
The rate of biofilm production between the retrieved *B. cereus* strains

All the tested *B. cereus* strains (*n* = 42) disclosed β-hemolysis on sheep blood agar. Moreover, 88.1% (37/42) of the recovered strains tested positive for biofilm production. Among the positive *B. cereus* strains, four isolates (10.8%, 4/37) were weak biofilm producers, ten isolates (27%, 10/37) were moderate biofilm producers, and twenty-three isolates (62.2%, 23/37) were strong biofilm producers (Fig. 2).

### Antimicrobial susceptibility of the retrieved *B. cereus* strains

**Fig. 3:**
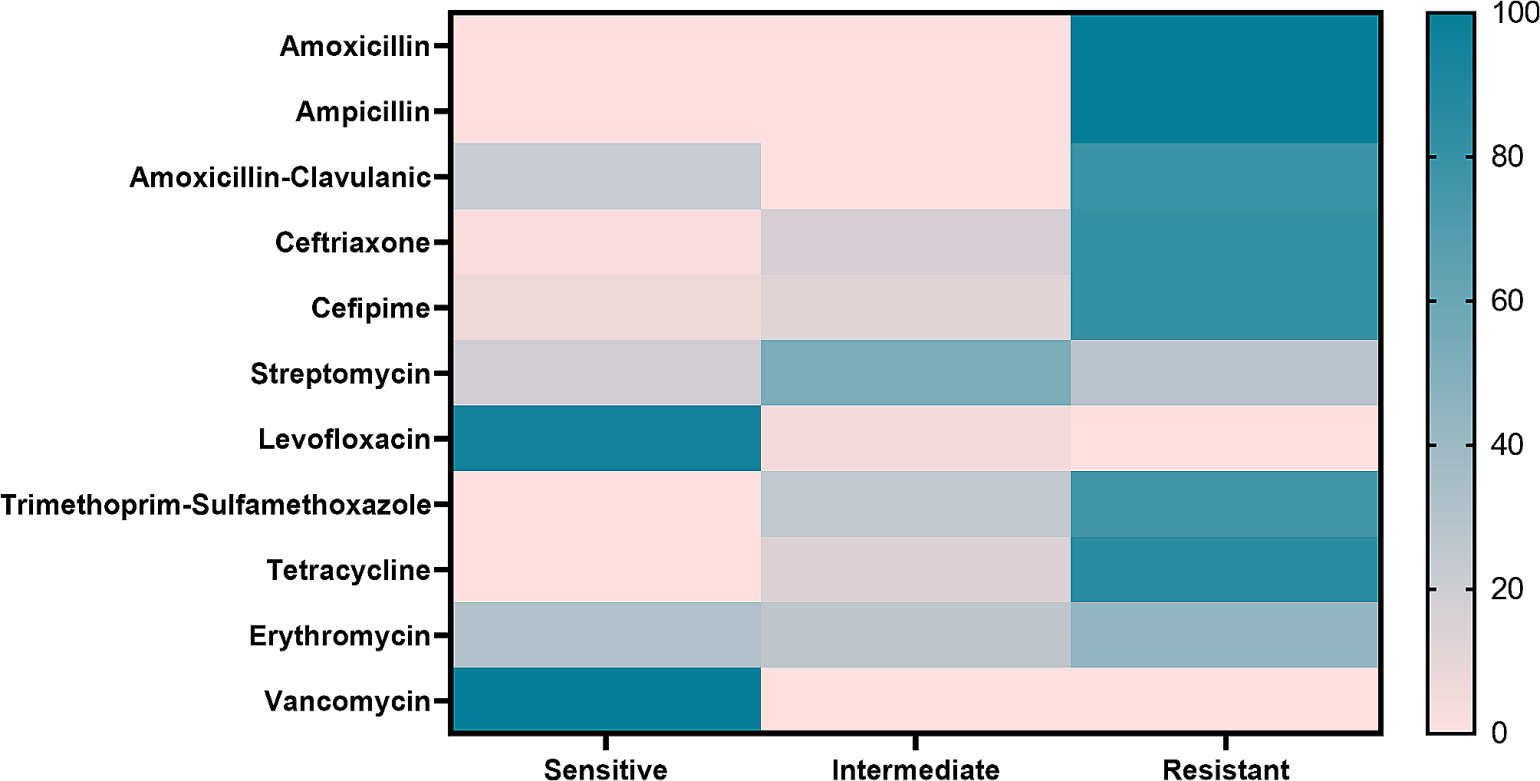
The antibiogram of the retrieved *B. cereus* strains. The bar (0-100) indicates the percentage of susceptibility

Resistance to antimicrobial agents among the *B. cereus* isolates was identified as follows: ampicillin and amoxicillin (100%, *n* = 42), tetracycline (85.7%, *n* = 36), cefepime and ceftriaxone (80.9%, *n* = 34), amoxicillin-clavulanic acid (78.6%, *n* = 33), trimethoprim-sulfamethoxazole (76.2%, *n* = 32), and erythromycin (42.9%, *n* = 18). Moreover, the recovered isolates were sensitive to vancomycin (100%, *n* = 42) and levofloxacin (95.2%, *n* = 40) (Table 3; Fig. 3). Statistically, the tested *B. cereus* strains revealed a significant variance in their susceptibility to various antimicrobials (*p* < 0.05).

**Table 3:** Antibiogram of the obtained *B. cereus* strains (*n* = 42)

Classes	Antimicrobials			Interpretation			
		Sensitive		Intermediate		Resistance	
		n	%	n	%	n	%
Penicillin	Amoxicillin	0	0	0	0	42	100
	Ampicillin	0	0	0	0	42	100
Tetracyclines	Tetracycline	6	14.3	0	0	36	85.7
Cephalosporins	Ceftriaxone	1	2.3	7	16.7	34	80.9
	Cefipime	3	7.2	5	11.9	34	80.9
Aminoglycoside	Streptomycin	8	19	22	52.4	12	28.6
Fuluroquinolones	Levofloxacin	40	95.2	2	4.8	0	0
Sulfonamides	Trimethoprim-Sulfamethoxazole	0	0	10	23.8	32	76.2
Macrolides	Erythromycin	13	30.9	11	26.2	18	42.9
Glycopeptides	Vancomycin	42	100	0	0	0	0
β -Lactam- β-lactamase inhibitor combination	Amoxicillin-clavulanic acid	9	21.4	0	0	33	78.6
*Chi-square* *p* value		213.77 *p* > 0.0001		94.105 *p* > 0.0001		94.693 *p* > 0.0001	

### Presence of toxigenic and antimicrobial resistance genes

**Fig. 4:**
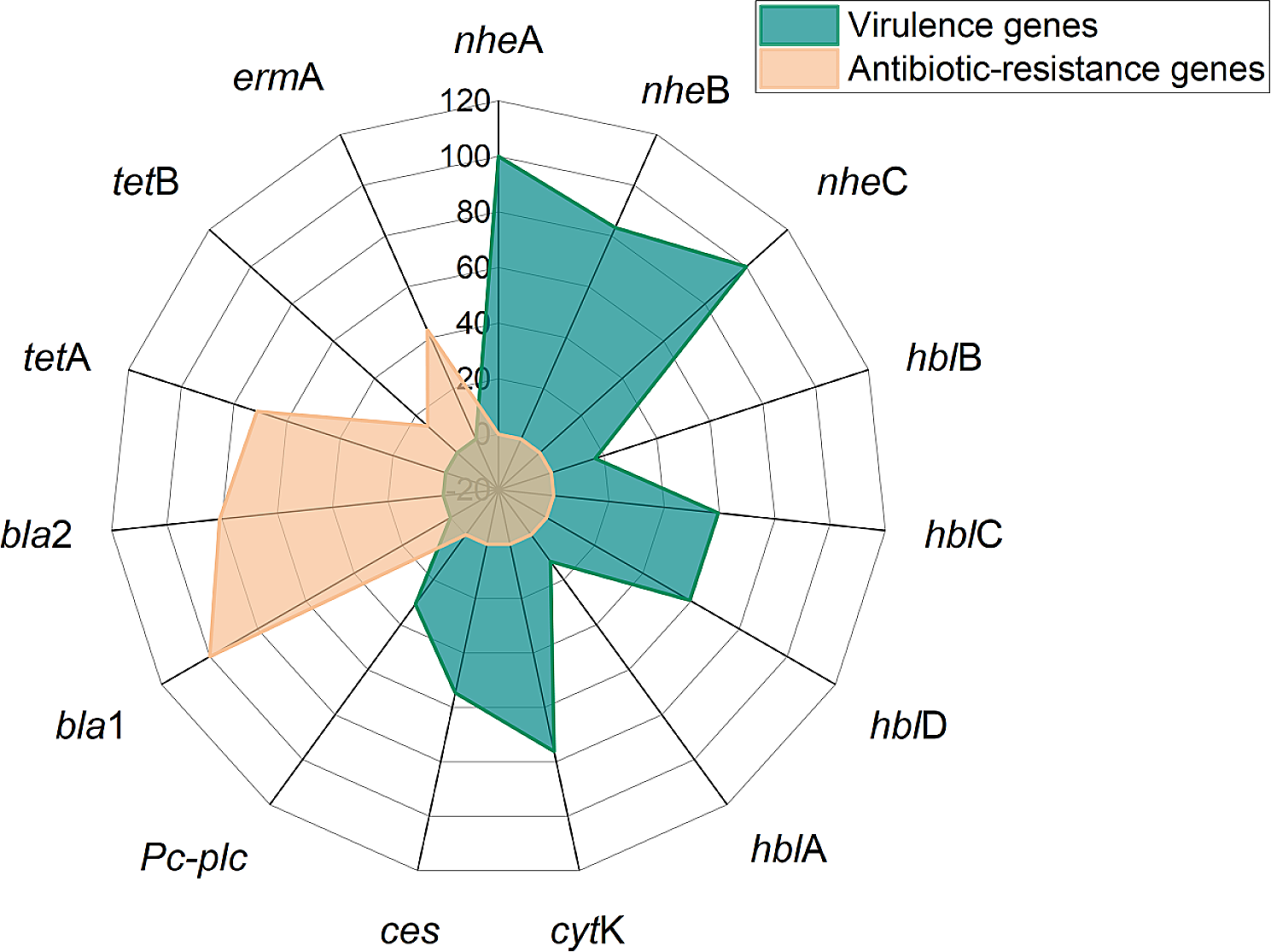
The occurrence of toxigenic and antimicrobial resistance genes in the retrieved *B. cereus* from food samples

Virulence-related genes were identified in *B. cereus* isolates as follows: *nhe* complex genes (*nhe*A and *nhe*C: 100%, *n* = 42, and *nhe*B: 83.3%, *n* = 35), followed by *cyt*K (76.2%, *n* = 32), *hbl* complex (*hbl*C and *hbl*D: 59.5%, *n* = 25, *hbl*B: 16.6%, *n* = 7, and *hbl*A: 11.9%, *n* = 5), *ces* (54.7%, *n* = 23), and *pc-plc* (30.9%, *n* = 13) virulence genes. Also, PCR evidenced that the tested *B. cereus* isolates inherited or acquired the *bla*1, *bla*2, *tet*A, *erm*A, and *tet*B antibiotic resistance genes with a prevalence of 100% (*n* = 42), 80.9% (*n* = 34), 71.4% (*n* = 30), 42.9% (*n* = 18), and 14.3% (*n* = 6), respectively (Table 4; Fig. 4). Statistically, a significant variance (*p* < 0.05) was noticed in the frequency of toxigenic and resistance genes in the recovered strains.

**Table 4:** Distribution of toxigenic and resistance genes in the obtained *B. cereus*

Type	Genes	n	%	Chi-square *p*-value
Toxigenic genes	*nhe*A	42	100	55.723 *p* < 0.0001
	*nhe*B	35	83.3	
	*nhe*C	42	100	
	*hbl*B	7	16.6	
	*hbl*C	25	59.5	
	*hbl*D	25	59.5	
	*hbl*A	5	11.9	
	*cyt*K	32	76.2	
	*ces*	23	54.7	
	*pc-plc*	13	30.9	
Antibiotic resistance genes	*bla*1	42	100	30.769 *p* > 0.0001
	*bla*2	34	80.9	
	*tet*A	30	71.4	
	*tet*B	6	14.3	
	*erm*A	18	42.9	

### Phenotypic and genotypic resistance patterns of the obtained *B. cereus* strains

**Fig. 5:**
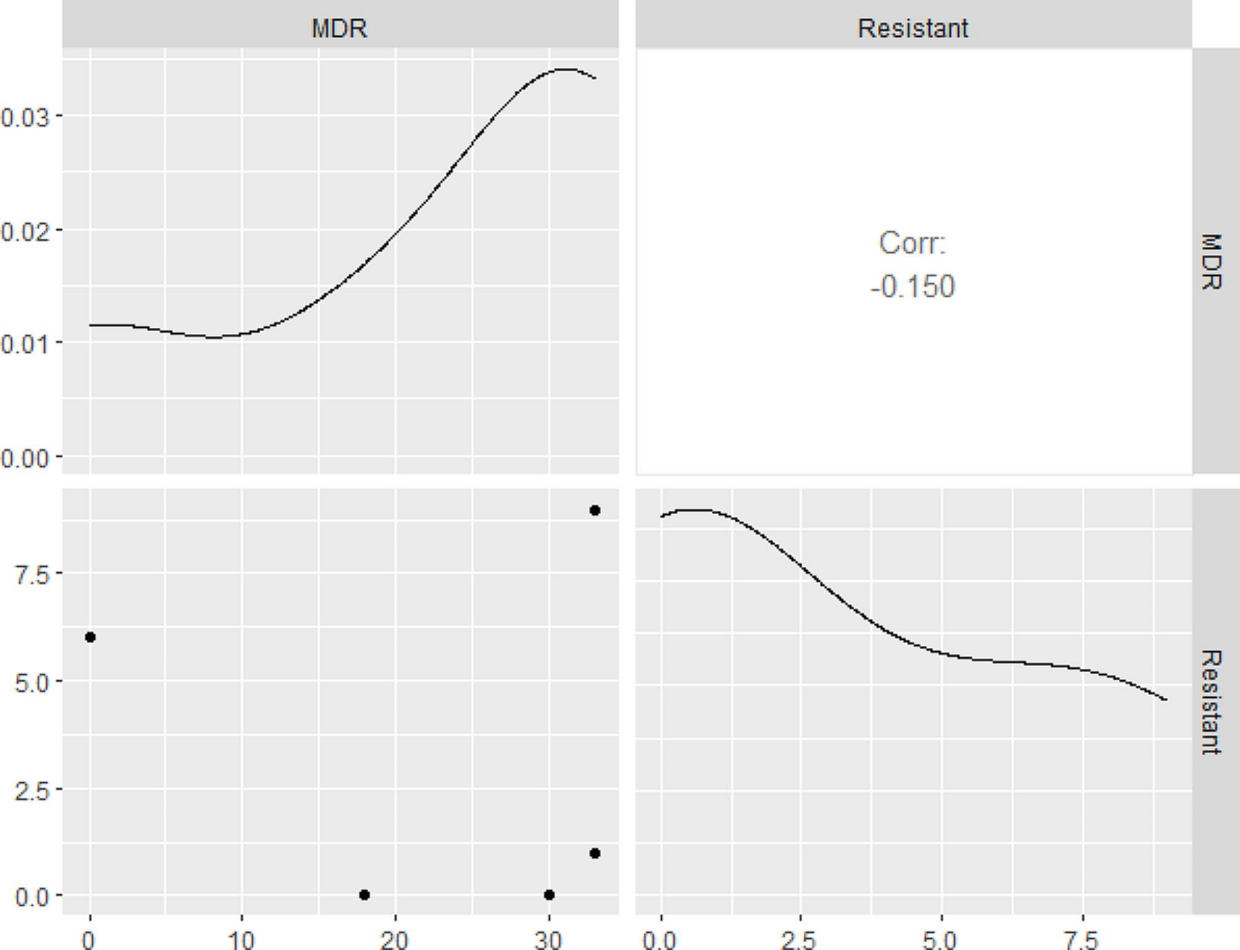
The occurrence of MDR among the retrieved *B. cereus* isolates from food samples

**Fig. 6:**
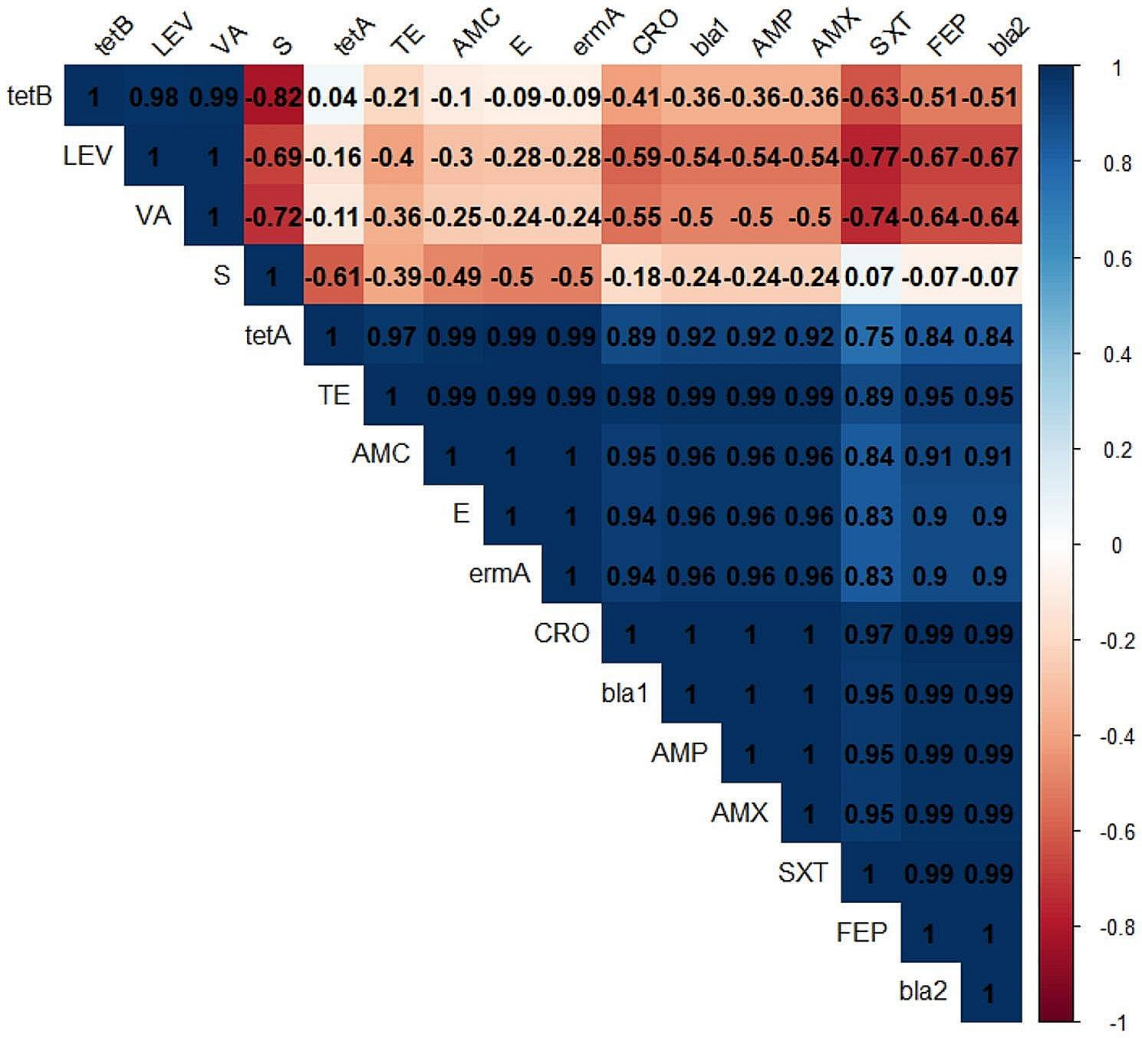
The correlation coefficient between the detected resistance genes and the involved antimicrobial agents (0.2 ≤ *r* ≤ 0.39: Weak, 0.4 ≤ *r* ≤ 0.59: Moderate, and *r* ≥ 0.6: Strong positive correlation)

Herein, 42.9% (18/42) of the examined *B. cereus* strains were MDR to eight antimicrobial agents (six antimicrobial classes) and encoded *bla*1, *bla*2, *erm*A, and *tet*A genes, while 28.6% (12/42) of the isolated *B. cereus* strains were MDR to eight antimicrobial agents (six antimicrobial classes) and inherited *tet*A, *bla*1, and *bla*2 genes. Besides, 7.1% (3/42) of the recovered strains expressed multidrug resistance to five antimicrobial agents (three antimicrobial classes) and encoded *bla*1 and *bla*2 genes, as clarified in Table 5; Fig. 5. In this study, the multiple antibiotic resistance (MAR) index values ranged 0.27–0.73 signifying that the obtained *B. cereus* strains were developed from high-risk contamination. Moreover, the correlation coefficient (r) was estimated between the identified resistance genes and the involved antimicrobial agents. Positive correlations were detected among *bla*1 gene, AMX, and AMP; *bla*2 and FEP; *bla*1 and CRO; *erm*A and E (*r* = 1 for each); *bla*2 and CRO (*r* = 0.99); *bla*1 and FEP (*r* = 0.99); *tet*A and TE (*r* = 0.97); *bla*1 and AMC (*r* = 0.98); *bla*2 and AMC (*r* = 0.91) as revealed in Fig. 6.

**Table 5:** Resistance patterns and resistance genes of the obtained *B. cereus* from food samples (*n* = 42)

*B. cereus*		Type	Resistance patterns	Antibiotic resistance genes	MARI
n	%				
18	42.9	MDR	**8 Antimicrobial agents/ 6 Classes**: AMP, AMX, AMC, FEP, CRO, TE, SXT, and E	*bla*1, *bla*2, *erm*A, and *tet*A	0.73
12	28.6	MDR	**8 Antimicrobial agents/ 6 Classes**: AMP, AMX, AMC, FEP, CRO, TE, SXT, and S	*bla*1, *bla*2, and *tet*A	0.73
6	14.2	Resistant	**3 Antimicrobial agents/ 2 Classes**: AMP, AMX, and TE	*bla*1 and *tet*B	0.27
3	7.1	MDR	**5 Antimicrobial agents/ 3 Classes**: AMP, AMX, AMC, FEP, and CRO	*bla*1 and *bla*2	0.45
2	4.8	Resistant	**3 Antimicrobial agents/ 2 Classes**: AMP, AMX, and SXT	*bla*1	0.27
1	2.4	Resistant	**4 Antimicrobial agents/ 2 Classes**: AMP, AMX, FEP and CRO	*bla*1, and *bla*2	0.36

Ampicillin (AMP), amoxicillin (AMX), amoxicillin-clavulanic acid (AMC), cefepime (FEP), ceftriaxone (CRO), sulfamethoxazole/trimethoprim(SXT), streptomycin (S), tetracycline (TE), erythromycin(E)

### The correlation between biofilm production and antimicrobial resistance

**Fig. 7:**
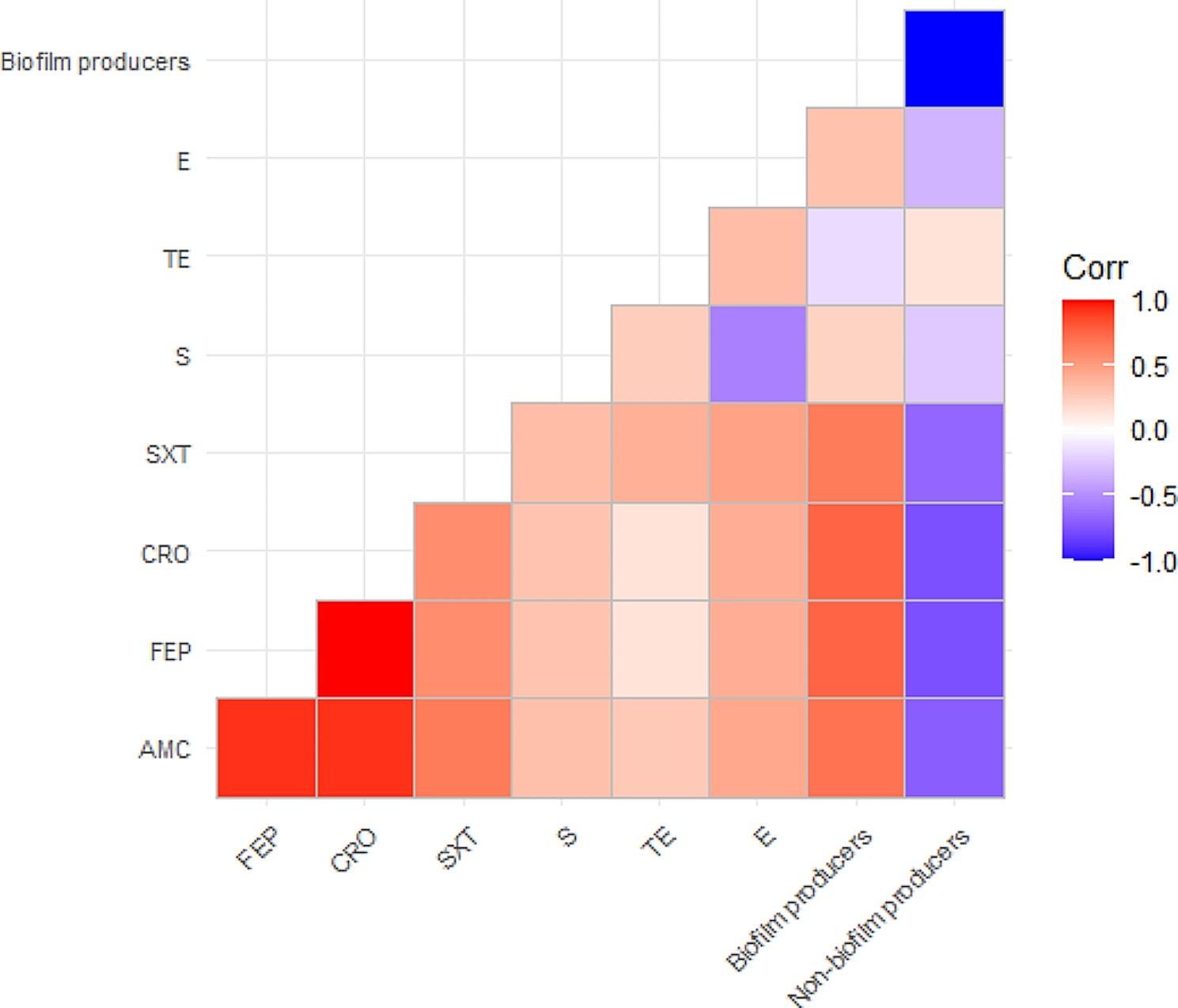
Illustrates the correlation between biofilm production and antimicrobial resistance

In the present study, the strong biofilm producers were resistant to eight antimicrobial agents. Besides, the moderate biofilm producers were resistant to 5–8 antimicrobial agents. Moreover, the weak biofilm producers were resistant to 3–4 antimicrobial agents, while the non-biofilm producers were resistant to 3 antimicrobial agents, as illustrated in Table 6. Statistically, a positive correlation was noticed between biofilm production and antimicrobial resistance (Fig. 7).

**Table 6:** The correlation between biofilm production and the antimicrobial resistance patterns

*B. cereus* (*n* = 42)		Resistance patterns	Biofilm production
n	%		
18	42.9	**8 Antimicrobial agents/ 6 Classes**: AMP, AMX, AMC, FEP, CRO, TE, SXT, and E	Strong
5	11.9	**8 Antimicrobial agents/ 6 Classes**: AMP, AMX, AMC, FEP, CRO, TE, SXT, and S	Strong
7	16.7	**8 Antimicrobial agents/ 6 Classes**: AMP, AMX, AMC, FEP, CRO, TE, SXT, and S	Moderate
3	7.1	**5 Antimicrobial agents/ 3 Classes**: AMP, AMX, AMC, FEP, and CRO	Moderate
2	4.8	**3 Antimicrobial agents/ 2 Classes**: AMP, AMX, and SXT	Weak
1	2.4	**4 Antimicrobial agents/ 2 Classes**: AMP, AMX, FEP, and CRO	Weak
1	2.4	**3 Antimicrobial agents/ 2 Classes**: AMP, AMX, and TE	Weak
5	7.1	**3 Antimicrobial agents/ 2 Classes**: AMP, AMX, and TE	Non-biofilm producers

## Discussion

*Bacillus cereus* is one the utmost predominant food-borne pathogens, resulting in severe food poisoning in humans. Herein, we intended to investigate the occurrence, *gro*EL gene sequencing, biofilm production, and resistance traits of emerged MDR *B. cereus* in meat and its products. Moreover, this work highlights the toxigenic and antimicrobial resistance genes.

In this study, all the obtained isolates revealed the characteristic phenotypic traits of *B. cereus* and disclosed coordination in their biochemical activities, as previously reported by Gdoura-Ben et al. in Tunisia [39]. Herein, the total prevalence of *B. cereus* in the inspected samples was 21%, where the highest predominance was detected in minced meat and beef meat. These findings signify that contaminated ready-to-eat food products could be a probable risk to human consumers. The higher prevalence of minced meat and beef meat, in comparison with luncheon, could be attributed to the heat treatment of luncheon during the processing stages, which reduces the potential *B. cereus* contamination [40]. A higher frequency of *B. cereus* (35%) was recorded in ready-to-eat foods in China; the prevalence was 34% in the cocked meat samples [41]. *B. cereus* is a ubiquitous pathogen, so open-air shops raise the potential for environmental pollution with *B. cereus* spores. Moreover, poor hygienic procedures during the processing and storage of various food products favor the contamination with *B. cereus* [42, 43].

Alarmingly, in this work, the mean *B. cereus* counts in the examined food samples exceeded the permissible limits reported by the Health Protection Agency [44] (should be less than 10^3^ CFU/g or mL). Moreover, Stenfors et al. [3] confirmed that lower doses of *B. cereus* in foods could result in severe food poisoning outbreaks in human consumers. Besides, the food is not suitable for human consumers if the *B. cereus* count is *>* 10^4^ CFU/g or mL [45]. The remarkably high counts of *B. cereus* in food products could be attributed to inadequate hygienic measures during food processing, contamination of equipment, inappropriate handling, bad storage conditions, and inadequate sterilization of equipment and machines [46].

The findings of this study showed that all the *B. cereus* isolates carried the *gro*EL gene. Wei et al. [47] reported that *gro*EL is a consistent diagnostic biomarker when compared with the *gyr*B gene to differentiate *B. cereus* from other pathogens in food products. The sequence analysis of the *gro*EL gene emphasized that the examined *B. cereus* strain revealed a notable genetic identity as well as cross-lineage with other strains derived from various geographical areas, such as *B. cereus* strain Q1 in China [48], *B. cereus* strain *B. cereus* strains JEM-2 and AH187 in the USA [49].

Remarkably, all retrieved *B. cereus* strains exhibited β-hemolysis on sheep blood agar. These findings agreed with those confirmed by Hwang and Park in the Republic of Korea [50]. Potent hemolytic activity is usually associated with foodborne *B. cereus* strains due to inherited factors [51]. In the current study, 88.1% of the recovered *B. cereus* strains tested positive for biofilm production (out of them, 62.2% were strong biofilm producers), suggesting the recovered isolates are highly pathogenic. Our findings agree with Osman et al. [52], who reported biofilm production in 83.3% of isolates (out of them, 33.3% were strong biofilm producers) obtained from different meat samples in Egypt. Strong biofilm producers are highly pathogenic and frequently resistant to phagocytosis, antimicrobials, and antiseptics [16]. The biofilms perform a substantial role in the binding of pathogens to the biotic and non-biotic surfaces that specify the positive correlation between biofilm production and the occurrence of infection. Besides, it reveals the potential public health threat of *B. cereus* in causing food poisoning to human consumers [53].

In this study, vancomycin and levofloxacin exerted potent antibacterial activity toward the recovered *B. cereus* strains from various examined food samples. These findings agree with those confirmed by Ikeda et al. in Japan [54], who reported that all the obtained *B. cereus* strains were highly susceptible to vancomycin, and only 10.3% were resistant to levofloxacin. Moreover, the obtained *B. cereus* strains were resistant to ampicillin, amoxicillin, tetracycline, cefepime, ceftriaxone, amoxicillin-clavulanic acid, and trimethoprim-sulfamethoxazole. Our findings agree with those highlighted by Savić et al. [55]. Also, a previous study in Egypt reported that 40% of the retrieved *B. cereus* isolates were MDR to eight tested antibiotics [19]. Besides, Yu et al. [41] confirmed that the recovered *B. cereus* isolates from food products in China were MDR to penicillin, cephalothin, ampicillin, cefoxitin, and amoxicillin-clavulanic acid. These outcomes confirmed the occurrence of MDR *B. cereus* in various food products, suggesting that ready-to-eat foods could be a main source of transmission of foodborne MDR *B. cereus* to human consumers [56]. The haphazard use of antimicrobials in the agriculture and health sectors favors the development of MDR strains [19].

Concerning the dissemination of toxigenic genes, our findings agree with the results confirmed by Tewari et al. in India [43]. Moreover, the combination *of nhe*A and *nhe*C genes was noticed in all tested strains, which agrees with the results reported by Fraccalvieri et al. in Italy [57]. In this work, all the obtained *B. cereus* strains inherited two or more enterotoxigenic genes, highlighting their public health significance as a causative agent of severe food poisoning in man, consistent with Owusu-Kwarteng et al. [58]. Foodborne *B. cereus* strains frequently encode one or more *hbl* complex genes [33]. Food poisoning caused by *B. cereus* is concomitant mainly with these virulence determinants: non-hemolytic enterotoxin (regulated by *nhe*ABC complex genes), the hemolysin BL (regulated by *hbl*ABCD complex genes), the cytotoxin K (regulated by *cyt*K), and the cereulide toxin (regulated by the *ces* gene) [9, 59].

In this study, the recovered *B. cereus* strains were MDR to eight tested antimicrobial agents (six different classes) and commonly carried *bla*1, *erm*A, *tet*A, and *bla*2 genes. Our findings are consistent with those confirmed by Fiedler et al. in Germany [6]. Moreover, the MARI values were 0.27–0.73 (> 0.2), worryingly highlighting that the retrieved strains resulted from high-risk contamination. The *bla*1 gene is mainly responsible for penicillinase enzymatic activity, whereas the *bla*2 gene is related to cephalosporins and penicillinase enzymatic activity [60]. Furthermore, the combination of *bla*1 and *bla*2 resistance genes is mainly responsible for the resistance of the β-Lactam-β-lactamase inhibitor combination [61]. Likewise, the resistance of *B. cereus* strains to tetracycline is mainly accredited to the *tet*A and or *tet*B gene, and their resistance to erythromycin is endorsed by the *erm*A gene. The existence of *tet*A and *tet*B genes in *B. cereus* emphasized the horizontal transfer of antimicrobial resistance genes from resistant pathogens to *B. cereus* [37].

Herein, a positive correlation was detected between the biofilm production and the antimicrobial resistance. Strong biofilm-producing *B. cereus* strains are highly virulent and usually resistant to disinfectants and antibiotics [16]. Biofilm-producing *B. cereus* is a highly adapted pathogen that could gain resistance to several antibiotics due to incessant disclosure to the antimicrobials, harboring or acquiring the resistance genes. Moreover, biofilm plays a vital role in drug resistance by delivering a proper environment for resistance determinant transfer [62].

In summary, this study highlights the presence of MDR *B. cereus* in meat and meat products, posing a significant public health risk. The contamination by *B. cereus* is common in minced meat and beef meat. MDR *B. cereus* isolates from food products often exhibit biofilm production and commonly harbor the *nhe* complex, *cyt*K, *hbl* complex, *ces*, and *pc*-*plc* virulence genes, and *bla*1, *tet*A or *tet*B, *bla*2, and *erm*A antibiotic resistance genes. Vancomycin and levofloxacin demonstrate promising antibacterial activity toward the retrieved isolates. The molecular assay is a reliable fundamental tool for screening emerging MDR *B. cereus* strains in meat and meat products.

## Data Availability

No datasets were generated or analysed during the current study.
